# Using AI to model future societal instability

**DOI:** 10.1016/j.futures.2025.103543

**Published:** 2025-02

**Authors:** Fanqi Zeng, Grant Blank, Ralph Schroeder

**Affiliations:** a Department of Sociology, University of Oxford, Oxford, United Kingdom; b Oxford Internet Institute, University of Oxford, Oxford, United Kingdom

**Keywords:** AI, Social forecasting, Societal dynamics Crisis, Data-driven modeling, Large language models

## Abstract

This paper develops a model that aims to pinpoint the future structural constraints facing a number of countries and the instability that may result from these constraints. The model uses existing datasets and extrapolates major patterns several decades into the future based on past patterns. Contrary to predictions of looming crisis in certain states by Turchin and others, the argument is that a more likely scenario is an increasing inability to cope with the combination of fiscal constraints that limit state revenue in the face of rising social spending. The paper is based on a four-way comparison between the United States, Sweden, India and China. These four cases provide a wide range of possibilities for comparative-historical analysis and forecasting. In the most likely scenario, a shrinking working-age population leads to a spending crisis in China and to social tensions in other countries. The paper makes three contributions: the first is to offer an alternative to Turchin’s prediction of political crisis in the US and beyond. The second is to extend predictions for societal instability beyond rich Western countries. The third is to demonstrate how our model can be compared with Turchin’s using AI tools.

## 1 Introduction

Research in computational social science (CSS) and artificial intelligence (AI) has grown sharply in recent years. One area where CSS and AI have been less prominent is in the understanding of long-term societal dynamics. An exception is the work of Peter Turchin, and we take his research about historical patterns and political crisis as our point of departure. His model is closest to our own, and so we can compare some of his findings to ours. Although our approach is quite different from Turchin’s, we share his aim of a social science where knowledge cumulates by means of formal modeling and these models are tested with data in order to forecast or explore potential futures. We argue that while using CSS and AI is currently rare in forecasting comparative-historical trends, there are potentially great gains to be made since data are abundant and hypotheses can be tested iteratively. These are two of the main pre-conditions for using AI or any other methodology to advance social scientific knowledge.

The paper starts by reviewing previous work, focusing on comparative-historical analysis and the cumulation of social scientific knowledge by means of computational modeling and large-scale datasets. Next, it compares two models of societal dynamics: the first is Turchin’s version of societies as complex systems which predicts state crises due to elite overproduction. We briefly present Turchin’s model, and then describe our alternative, which focuses on the limits to post-war welfare states and constraints on state spending. The two models have quite different scope conditions, but we argue that the main predictions can nevertheless be compared. Whereas Turchin focuses on instability in the sense of political crisis and his main example is the United States (US) (though he applies the model across history), our focus is on instability and on the post-war period and coming decades in four countries. We discuss the limitations, potential extensions, and implications in the conclusion.

## 2 Background and previous work

There is much discussion on how computational approaches and AI are changing knowledge production. Salganik (2019) has provided an overview of the pitfalls and opportunities of these approaches for social scientists, and Lazer et al. (2020) have laid out an agenda for computational social science (CSS). There are many areas where these tools are being developed rapidly; here, we focus narrowly on analyzing historical patterns to explain and forecast the causes of political crises. This is an area of social science where cumulation (Collins & Sanderson, 2016: 10–12) can be said to be taking place, and this leads us to the definition of AI we use here, which is machines that learn to learn. This is a pragmatic and narrow definition, far from the idea of an ‘artificial general intelligence’ (AGI) that is about machines that think. Here we are simply concerned with computational tools that cumulate social science knowledge, that learn to learn, with machines driving these advances. This obviously sets these new approaches apart from regression models, which are single-shot attempts to identify causal relations: AI-driven approaches should, in contrast, set up workflows that allow machines to iteratively refine and extend hypothesis testing (Mökander & Schroeder, 2022).

There have been other attempts to predict future economic and political patterns, and especially forecasting global economic futures. We can take here as an example the recent report from Goldman Sachs (2022) to illustrate how our model differs. First, we can note that the report includes many countries but not Sweden. That is because the Goldman Sachs is interested in the largest markets. China, India, and the US are included, as are some projections for big European economies like Germany. Second, apart from economic growth and demographic projections, it includes five other ‘conditions for growth’ (Goldman Sachs, 2022: 15–16): institutional quality, openness to trade, education, macro stability, and infrastructure quality. We simply note here that average tax levels are not included and this is because, as the report rightly notes, ‘there are rich economies with high taxes, poor economies with low taxes, and vice versa’. (Goldman Sachs, 2022: 16, note 13). We also note that these five ‘conditional’ factors for growth are part of a proprietary model (Goldman Sachs, 2022: 36); the methodological procedures of which are not public. The report adds that these conditional factors do not add much to the model’s predictive power (Goldman Sachs, 2022: 25, 36). In short, the report mostly focuses on economic growth and demography, leaving all else to one side.

The results of the report are projected to the year 2075. These include several predictions worth attention. First, the decline in the working age population, particularly in China, will likely to be a significant ‘drag’ on growth. Second, it predicts that overall the difference between emerging and developed market economies will decline, continuing a 20-year convergence pattern. However, this contrasts with growing inequality within countries. For this reason, the report points to the dangers of increasing populism and economic nationalism that could endanger future growth. Another future danger is climate change which ‘requires economic sacrifices and globally coordinated response, both of which will be difficult to achieve’ (Goldman Sachs, 2022: 7). But the main aim of the report is not these dangers but rather growth projections, and here we can note the predictions that by 2075, India will catch up with the US economy in overall size and China will do so around 2035 (Goldman Sachs, 2022: 20). On a GDP per person basis, the US will still be far ahead of both countries in 2075: US $132,200 versus China $55,400 and India $31,300, though that would represent a significant catchup compared to today (in 2020: US $64,800, China $10,900, India $2000). For our purposes the main point to keep in mind is simply that China’s aging population, and likewise the age structures elsewhere, will play a decisive role. As we shall see, an aging population is indeed a major constraint.

Another set of researchers who are interested in long-term social and economic projections are climate change scientists (Leimbach et al. 2017). They have developed a shared socio-economic pathways approach whereby different scenarios are spelled out based on different assumptions about whether economic growth will converge or diverge in different regions, also a key factor in the Goldman Sachs report (Goldman Sachs, 2022). This is particularly important for climate change because these alternatives imply quite different magnitudes of world growth until 2100. These different forecasts in turn have different implications for sustainability. For example, if per capita income is compared for the US, China, India, and sub-Saharan Africa until the year 2100 (Leimbach et al. 2017: 221, fig 5), then there would be quite different consequences depending on whether these four regions or countries converge by 2100 or if they continue to diverge. These models’ central components include the working-age population, total factor productivity, technological catchup and education levels.

The main point to take away from these analyses for our purposes is that there are several projections of world GDP growth based on different scenarios. These are not AI or CSS models in the sense that we consider ours and Turchin’s as such, and in the case of the Goldman Sachs model, it is also proprietary and so not replicable and thus not cumulative either. The Leimbach et al. model is replicable, but it aims at forecasting rather than our goal of cumulative model-building. Further, climate change researchers are mainly concerned about what growth will mean for the planet rather than for the economic well-being of populations or social instability (though the two are, of course, connected). Again, for climate change researchers the health of the planet depends on convergence versus divergence, and, as we saw, the Goldman Sachs report (Goldman Sachs, 2022) forecasts convergence between developed and emerging market economies. Divergence means lower growth among countries in the Global South and less impact on global warming. The key point for following analysis is that we can think about what the two extremes of convergence versus divergence imply for overall world growth within certain upper and lower bounds. These two studies are mainly about economic futures whereas Turchin (to be discussed shortly) is mainly interested in state breakdown. Our analysis tries to connect economic and political futures.

Both projections agree that the size of the working-age population, for example in China, will be key. This population segment is crucial because they pay the taxes that support the non-working young and elderly. The larger the proportion of the population that is of working age, the greater the tax base and the more flexibility it gives the government to provide social spending. The Goldman Sachs (2022) and Leimbach et al. (2017) models treat education in terms of skills for growth, whereas Turchin, as we shall see, treats education as leading to a crisis among elites. Our analysis leaves out education altogether, but like Turchin, we are interested in economic strains on the state. In analyses such as that by Goldman Sachs (2022) and Leimbach et al. (2017), the state plays little role, and it is not theorized explicitly. In addition to the relative size of the working-age population we also assume that the state could have severe political difficulty increasing revenue by increasing taxes, and that rates of growth during the Western post-war ‘belle epoque’ of growth and in the Indian and Chinese heady years of growth in 1990s to 2010s are unlikely to be sustained. We are particularly interested in how the downturn in rates of growth and increasingly aged population will affect the state’s ability to provide growing welfare services needed by the rising elderly population.

### 2.1 Turchin’s model

Like many social scientists, Turchin’s ideas have evolved over time and he has presented them in different formats which also cover different countries and time periods. But the core idea, that societies go through periods of stability and instability or crises, is a common preoccupation in his writings. Turchin sees societies as complex systems with various feedback loops that can be modeled. He analyses these systems over long time periods and finds persistent cyclical patterns. Central to these patterns is what he calls the ‘wealth pump’, whereby the wealth from economic growth is redistributed. But during certain periods, resources can be scarce relative to the size of the elites that seek to gain their share of wealth – he calls this condition ‘elite overproduction’. During these periods, some groups that aspire to become elites find their path blocked. This leads to the rise of counter-elites, which leads to political crisis, such as that currently experienced in the US due to the rise of Trumpism. For Turchin, Trumpism and the potential for violence during a period of political instability is just one example among many in history in which this pattern where a weak ‘wealth pump’ leading to ‘elite overproduction’ has resulted in political crisis.

There is no need to reproduce Turchin’s various accounts in detail here, or the graphs and data he uses to support his arguments. We are mainly concerned with his ideas about the current period of instability. For our purposes, it will suffice to say where we agree and disagree with his ideas, and whether we use the same data sources or different ones. Turchin summarizes his model in his most recent book ‘End Times’ like this: When a state, such as the United States, has stagnating or declining real wages … a growing gap between rich and poor, overproduction of young graduates with advanced degrees, declining public trust, and exploding public debt … the data pointed to the years around 2020 when the confluence of these trends was expected to trigger a spike in political instability. And here we are.’ (Turchin, 2023: xi). Turchin made this prediction about the rise of political instability in the US and Europe already in 2010 (Turchin & Korotayev, 2020). But Turchin argues that such a model applies throughout the history of complex societies (Turchin, 2016, 2018), and in ‘End Times’ has also applied his ideas about state breakdown to other times in American history (the Civil War) and to ancient Rome and various cycles of violence in France and England and China (especially the Taiping rebellion).

There are several points where Turchin’s and our model could agree: first, growing inequality which produces ‘immiseration’ among the non-elites and will likely to lead to political tensions. Both models could be described as a ‘demographic-structural’ theory or model. Third, we agree that Trump’s presidency and the rise of populist parties in Europe constitute a major destabilizing shift in politics. Note, however, a pair of cautions. There is considerable disagreement about the causes of the rise of populism (Judis, 2016; Mudde & Kaltwasser, 2017; Norris & Inglehart, 2018) and others have argued (Schroeder, 2013) that the rise in Western populism is a result of a much longer-term trend of the ending of an era of post-war growth in the mid-1970s and 1980s (for example, the downturns in revenues/expenditures during these periods in Fig. 2 below). Fourth, on a more general level, we agree with Turchin’s aim to put history and social science disciplines on a more scientific and computational footing and to attempt forecasting or prediction. On a general level, however, we disagree with Turchin’s approach to complexity science: this approach is based on ‘non-linear’ feedbacks (2016: 21). It is a more complex model, but at its heart it models the relationship between population growth, productivity, and state capacity. Our approach is more mechanistic and linear but it looks at similar variables and examines similar relationships.

## 3 Modeling future instabilities

We provide some global figures but our primary focus is on four countries for three reasons: one is that only nation-states can provide indications of instability apart from those caused by geopolitical factors such as wars, and these countries provide a wide, valuable range of alternative scenarios. The second is that these four are often compared, though more often pairwise (Pontusson, 2005; Bardhan, 2012) since they provide not only high variation among otherwise similar cases but also extremes among the varieties of capitalism (Hall & Soskice, 2001) and models for the Global South in the case of India and China (Huang et al., 2011). Third, three of the country cases (China, India, and the US) have global implications, with Sweden mainly a comparator for the high-income US: we could have added other countries and future research will do so, but four also keeps the model from being too complex to be instructive for our purposes.

### 3.1 Data sources

We draw population data from the World Bank (https://databank.worldbank.org/source/population-estimates-and-projections/), series: ‘Population ages 15–64 (% of total population)’, time: 1960–2050. The World Bank draws data from the United Nations World Population Prospects (2022 revision, see WPP, 2022).^1^ It is worth calling readers’ attention to the fact that these projections are sensitive to the assumptions made about fertility, mortality and net migration. The UN (WPP, 2022) discusses the implications of this in more detail. The working age population is generally defined as people between the ages of 15 and 64. This is conventional and it has been historically broadly accurate, but we recognize that many people in those ages are in education or otherwise not in the labor force; for example, caring for children or the elderly. In addition, some people over age 64 continue to work. Government expenditure and revenue data are from the IMF DataMapper (https://www.imf.org/external/datamapper/datasets), ‘Public Finances in Modern History’ (range: 1800–2021) and the April 2023 Fiscal Monitor (range: 1990–2028). We combined these two series by using the data between 1960 and 2021 from the ‘Public finances’ series and the data between 2022 and 2028 from the ‘Fiscal monitor’ series to create a single series from 1960 to 2028.

### 3.2 Working age population trends

**Fig. 1:**
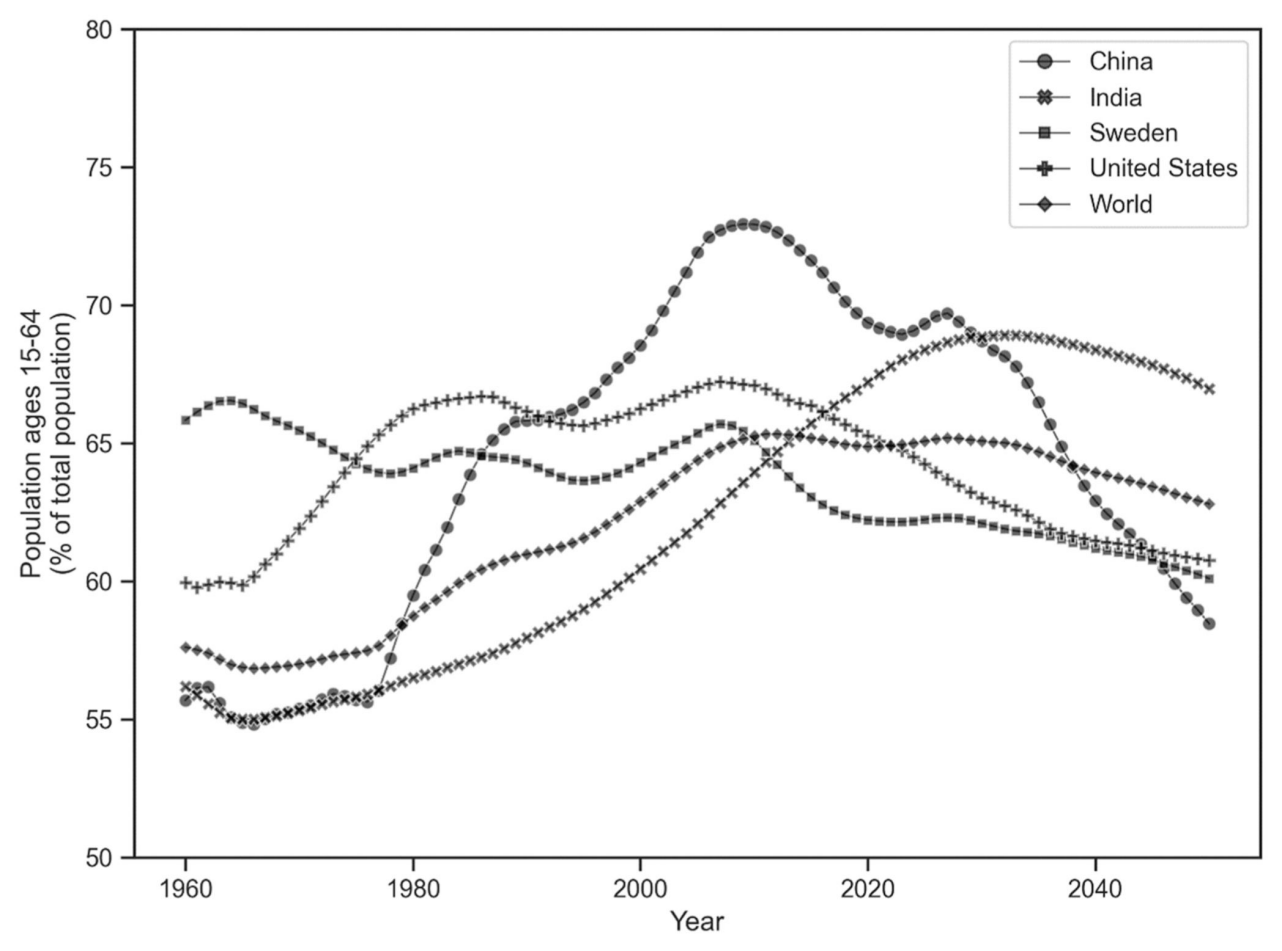
Working age population as a percent of total, 1960–2050. Note: Working age population is defined as ages 15–64. Percentages after 2021 are projections; see Data Sources section for details.

Fig. 1 shows the working age population (ages 15–64) as a percentage of the total population for the 90-year period from 1960 to 2050 for four countries plus the entire world. For all four countries the projected working percentage of the population is expected to decline, although the extent of the decline varies. The Swedish and Indian declines are expected to be relatively small, about two percentage points. Because of strong immigration, the United States shows at most a small decline of about four percentage points; other sources disagree. For example, according to the Congressional Budget Office, the American working age population is projected to grow by 0.2 % per year from 2023 to 2053 rather than declining, see https://www.cbo.gov/publication/58612. The projected decline in the Chinese workforce is dramatic. From its peak in 2010, the decline is about 15 percentage points to 2050, which has important implications for the future economic and political instability that we describe below.

### 3.3 Our model for possible futures

The decline in the working population poses problems for all four countries, especially for China because its decline is so large. The core problem is how governments can continue supporting the non-working population, particularly the growing elderly. To protect aging people, the government must spend more on pensions and health care, but this creates a budget problem because the working age population, hence tax revenue, is falling.

**Fig. 2:**
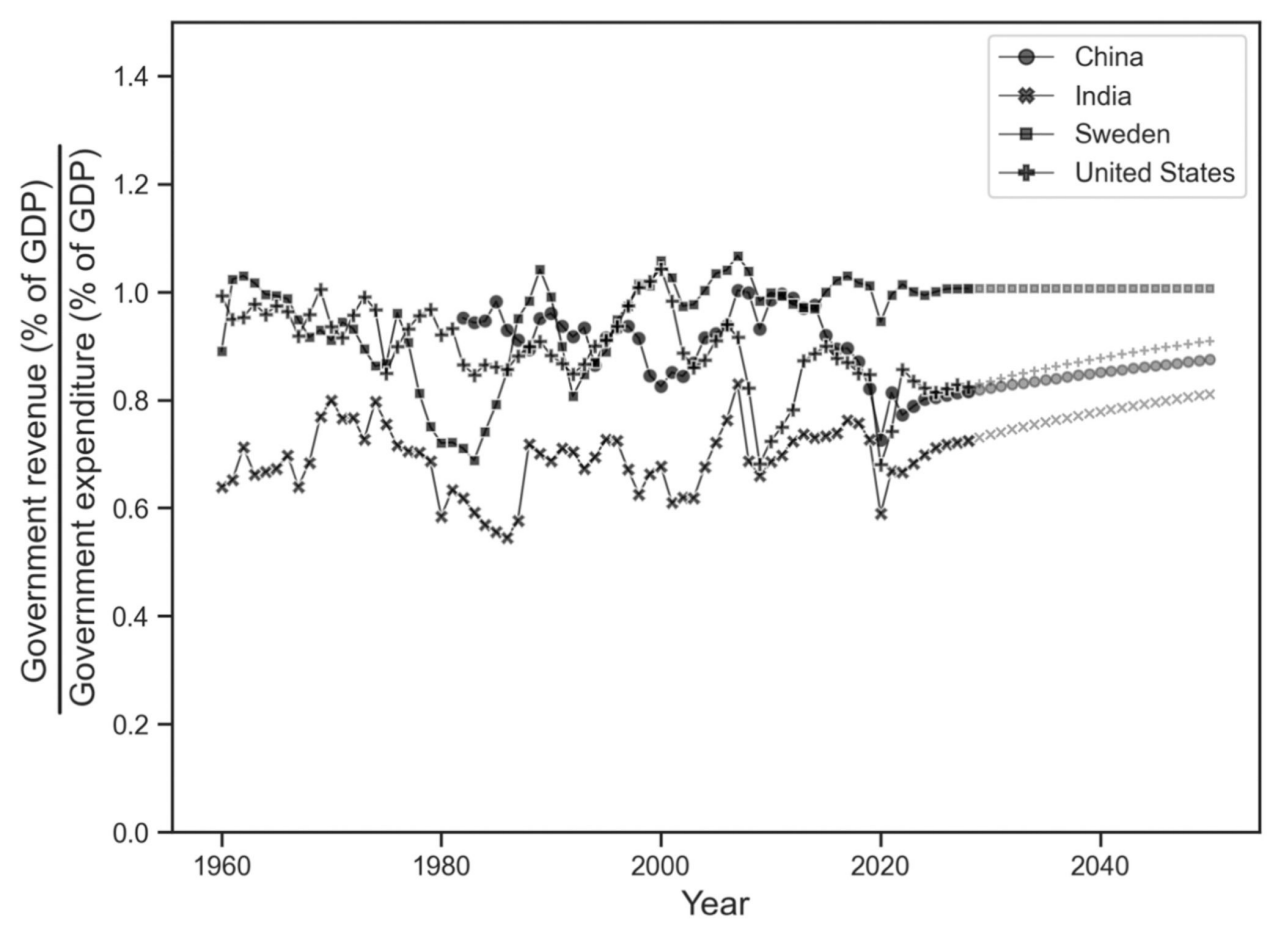
Government revenue as a proportion of expenditure. Note: Numbers after 2021 are projections. For 2022–2028, the World Bank used official government projections. The numbers after 2028 are our projections.

Government expenditure must ultimately balance revenue. If the government runs a deficit the shortfall must be made up by borrowing from the domestic private sector or foreign investors. If deficits continue, interest payments will rise, and larger portions of the budget must be devoted to paying for previous deficit spending. Continued deficits raise the total indebtedness of the government. Eventually investors begin to suspect they are at increasing risk of not being repaid and they demand a higher interest rate or simply refuse to buy additional bonds. These difficulties recently crystallized during the global financial crisis of 2007–8 and its aftermath. Fig. 2 plots the ratio of revenue to expenditure for all four countries from 1960 to 2023. There is considerable variation for all countries reflecting their specific circumstances.

We discuss each country in turn: Sweden is a developed welfare state with a tradition of stable government and a population with high income. It has generally had a balanced budget for the entire period except for a financial crisis in the early 1990s. India became the most populous country in the world in 2023. It has a stable democratic government, though recently tending towards majoritarianism (Jaffrelot, 2021), that has been pursuing market-led policies under Prime Minister Modi. It has been running a consistent deficit, but its nominal GDP growth rate has been 7–9 % in recent years, which has increased tax revenues. It has a high domestic savings rate, and has been able to fund the deficit primarily from domestic sources.

For many years China has been in an enviable economic position with large numbers of skilled people entering the labor force and rapid movement of people from rural areas to cities. The increase in the working-age population provided the revenue to sustain largely balanced government budgets. Recently several trends have begun to limit Chinese government spending. Following the wildest property boom in history the bubble burst, large government bailouts are needed, and defense spending has risen. China’s working-age population peaked in 2010 and its total population fell in 2022 for the first time since 1961, meaning the supply of new workers is declining. Offsetting the decline in the labor force is the possibility of productivity improvements. China is still playing catchup in the shift to a knowledge-based economy (Lei, 2023: 12–14), which gives it considerable room to improve worker productivity. China has two factors in tension: a declining labor supply and the need for increasing productivity. How they will balance is not clear.

The United States has run government deficits for all but a few years since 1960, but the US is a special case. It is different because the dollar is a world-wide reserve currency. Investors in other countries buy US government bonds because they are believed to be the safest financial instrument available. As long as foreigners are willing to continue to do this, American government revenue is not as constrainted by the size of the labor force and the government can finance deficit spending without the same consequences as other countries.

In Fig. 2 we used the last three years of data to project the ratio of government revenue to expenses to 2050 linearly. These projections assume there will be no large, unpredictable, exogenous shocks like another pandemic, financial crisis or a major war. We note that recent years for all four countries have been relatively stable so the projections form generally horizontal lines. Of course, the actual values will not be as neatly smooth as the projections. The value of the projections is that they illustrate a baseline scenario from which there will be inevitable deviations. There is information in the baseline scenario, which we discuss below.

Simple linear projections like this do not show major changes in the balance of revenues and expenses. For a politically stable, wealthy country like Sweden this is probably a reasonable estimate of its future prospects. For India the declining working population will reduce tax revenues. This implies some combination of increased productivity, increasing taxes or additional borrowing to meet the welfare costs of an increasingly elderly population, but the increase may be manageable because the projected labor force decline is small and India, with a large number of English-speakers, is benefitting from the movement of foreign investment away from China. This is likely to increase productivity.

The United States is most likely in a position similar to Sweden. It is wealthy and stable so it can afford to spend more on care for the elderly. Furthermore, its unique position where the dollar is the world-wide reserve currency means that budget deficits are not as serious a problem as they are for other countries. It is important to remember that the main American government pension fund, Social Security, will no longer meet all of its obligations to pay beneficiaries in the 2030s unless current taxes or benefits change. Up to now the political will has not been present to make these changes.

China is a distinctive case. As already mentioned, for decades the Chinese economy benefitted from large numbers of skilled people entering the labor force coupled with rapid movement of people from agriculture to cities where they were available as an inexpensive labor force. These trends have changed in recent years. The future rapid decline in the working age population will reduce tax revenues which will make it hard for China to find the additional money needed to provide pensions and health care for its rapidly aging population. Furthermore, the compulsory basic pension “will be in deficit by 2028 and run out entirely by 2035” (Economist, 2023). China is growing elderly before it becomes wealthy.

### 3.4 Using AI to test models

**Fig. 3:**
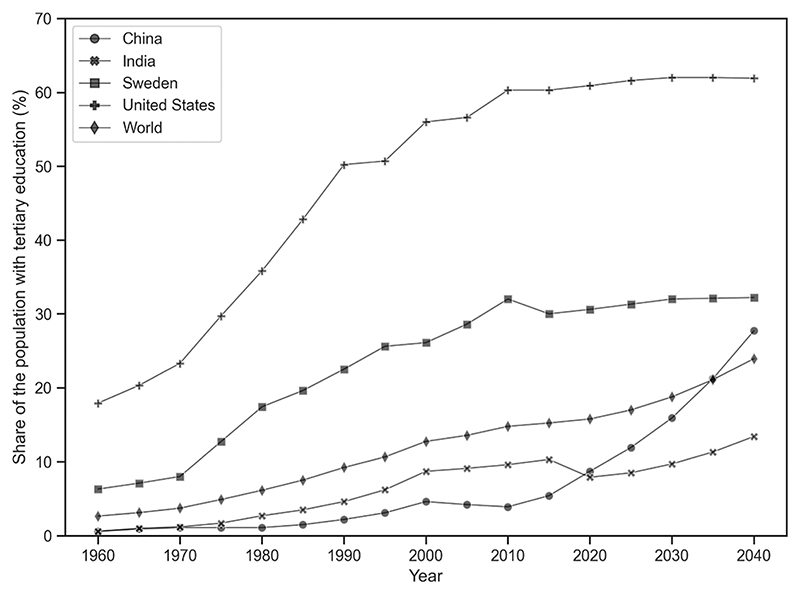
Proportion of population in tertiary education, 1960–2040.

So far, we have used some basic statistics and extrapolations to highlight future instabilities. We can now compare our findings to Turchin’s. Turchin predicts social instability due to elite overproduction in the US and elsewhere. But elite overproduction is an even greater danger in China over the longer term. For example, the proportion of the population with tertiary education (see Fig. 3) has a steep upward trajectory. The increase in university-level graduates who face a future without the requisite employment opportunities or reward in China would, according to Turchin, constitute a dramatic overproduction of elites. China is, of course, an authoritarian state, so the possibilities of crisis due to over-educated elites producing rival factions that engage in conflict over control of the state are different from those in the democratic US. Still, according to Turchin elite overproduction should also lead to severe social instability in China unless there is severe repression of a growing highly educated elite combined with increasing economic inequality.

Fig. 3 reproduces the data on the percentage of the population aged 25–65 years who have either completed or partially completed tertiary education from 1960 to 2040 using Our World in Data (https://ourworldindata.org/grapher/share-of-the-population-with-completed-tertiary-education?tab=table).

We have also argued that the need for additional state spending will likely lead to social instability, with a greater likelihood in some cases (China) than others (Sweden). But using data for state spending and higher education levels, an AI model should be able to learn which of these scenarios – state spending pressures or elite overproduction pressures – are causing greater social instability. The two models can be counterposed and tested for different time horizons. AI tools can answer queries (or ‘prompts’) such as those we have discussed in this paper. In our example, we use ClaudeAI (www.claudeai.com) because it can read and analyze the dataset. ClaudeAI is only one of a number of AI models that have recently become available: there are many more. Many are ‘large language models’ (LLMs) that have recently come to public attention through models like ChatGPT. We could have used other models here, but this is a fast-developing area of research, and it is not clear yet how best to assess their accuracy (for an early effort, see Rosenbusch, Stevenson, & van der Maas, 2023).

If we give the data that we used in our analysis to ClaudeAI (accessed on 7.12.2023), using the following prompt, we get the reply below:

Prompt:

‘Based on the expenditure and revenue data and patterns from China, India, Sweden, and the United States from 1960 to 2021, please use the working age population data to predict revenue and expenditure for the four countries until 2050.’

Reply:

‘Based on the historical data and trends, as well as taking into account the projected changes in working age population shares, here are my predictions for government revenue and expenditure as a percentage of GDP for China, India, Sweden and the United States from 2022 to 2050:

China Revenue: Will decline gradually from 26 % in 2020–23 % by 2050

Expenditure: Will decline from 35 % to 31 % by 2050

Drivers:

- Working age population share projected to decline steadily from 67 % to 61 % by 2050
- As population ages, spending needs may rise but revenue capacity will go down
  India Revenue: Will increase gradually from 20 % in 2020–25 % by 2050
  Expenditure: Will increase from 28 % to 31 % by 2050
  Drivers:
- Working age share will continue to edge up slowly to around 69 % by 2050
- Fast growth should drive higher formalization and tax capacity over time

Sweden Revenue: Will be stable in the 47–50 % range

Expenditure: Will be stable around 49–52 % range

Drivers:

- Working age population declining but ongoing need for social spending
- Revenue and expenditure levels already among highest globally

United States Revenue: Will remain steady at around 30–32 % range

Expenditure: Will remain steady around 34–36 % range

Drivers:

- Working age population share quite stable, although aging may increase costs
- No major upside or downside factors expected in revenue or expenditure

In summary, the patterns are largely based on working age demographic trends interacting with the existing scale and structure of the four economies. Let me know if you need any clarification or have additional questions!’

This is a simple prompt or question, and ClaudeAI provides a simple answer that overlaps with ours. We should also note that this model is black boxed in the sense that the parameters that it uses to respond to queries are unknown and not knowable. This is a characteristic shared by all current LLMs. There are large language models that are open source (e.g., Meta’s Llama 3, also see Bommasani et al. 2024), but this refers to the computer code that is applied to the text data to create the model. The model parameters, derived from the text data are unknown and not knowable. The openness or otherwise is currently a major debate in AI development, which is related to many issues, including intellectual property rights of the data sources but also the harm that could be done by open-source AI models if they were used to develop bioweapons and the like. The concerns about harmful use do not apply to our model in the domain of social forecasting, but the problems of black boxed models and lack of reproducibility will remain even if researchers are working towards solutions (e.g., for psychology, see Demszky et al. 2023). In the future, we may be able to make use of more transparent (non-black boxed) models, but that will depend on AI development.

There are several ways in which this analysis could be made more sophisticated. This prompt could be ‘fine tuned’ (using more than one ‘shot’, to use the language of model developers); that is, the question wording could be changed to ask for a comparison of the trajectories of the four countries, to add more data or more countries, or to make other specifications or modifications to the prompt. The same could be done for Turchin’s or other models, and then the AI tool could be asked to compare them. Another possibility would be to ask the AI tool to query online resources to see if other comparable research results could be brought to bear on the question. In this way, an ongoing learning process could be implemented, whereby the results from the models are iteratively checked against each other and against the data. This would entail putting the components of the models (ours, Turchin’s and others) into comparable formats, a task which could also be assigned to AI tools. We leave these ideas for future work: within the scope of this paper, we have laid the groundwork for implementing them.

Table 1 summarizes the predictions from us and ClaudeAI.

## 4 Conclusions and outlook

We disagree with Turchin that Trump and European populism constitute a ‘crisis’, though he is primarily concerned with political instability in the US. Some have argued that populism is not as much of a crisis as it may have seemed, though there is disagreement on this (Przeworski, 2019; Bartels, 2023). In any event, in line with our model, we conceptualize the growing force of populism as a gradual political shift resulting from the tensions we have identified. We agree with Turchin’s assessment of the US: Democrats have come to be seen as the party of elites and Republican support has shifted to white ‘working class’ supporters, reversing previous alignments. This diagnosis is shared by many others (Lind, 2020). Similar dynamics can be found in Sweden (Schroeder, 2023a) or Denmark, the example favored by Turchin, or other Nordics.

One place where disagreement is unclear, and which would need to be aligned to carry out the testing via AI models that we have briefly sketched, concerns when Turchin regards the recent crisis as originating: his model is based on cycles of crises of political instability throughout history. Unlike Turchin’s model, ours cannot cover longer periods because the welfare state hardly existed before the 20th century, and the most severe effects of the declining working age population are in the future. How the welfare state came about during the New Deal in the US and the creation of the ‘people’s home’ (folkhem) in Sweden – and subsequently in India and China - must be left to one side here for reasons of space (but see (Schroeder, 2021)). However, a crucial point is the post-war peak in income equality (see, for example, Scheidel, 2017; Mann & Riley, 2007) and recent increases in economic inequality (but see Auten & Splinter, 2024 for an argument that inequality has not increased).

Further, when we talk of instability we mean – not crisis, as for Turchin – but rather conditions that lead to a major turn in political direction or political tensions, which may increase gradually, depending on how the political system responds to the changed conditions. Turchin focuses on trend reversals or ‘tipping points’ (Collins, 2022: 54–65) where gradually increasing fiscal constraints leads to a major political shift and state breakdown: the two are different and so perhaps difficult to compare because they point to crises of different types: sudden and irreversible in the one case, and slow and subject to amelioration in the other. Another place where our analysis is similar to Turchin’s is that we primarily focus on the relations between economy and politics. Yet we differ in that Turchin conceptualizes this as elites versus commoners, while we see this in terms of how state elites respond to citizen welfare demands (Schroeder, 2021). Turchin focuses on elites and top-down economic interests in a situation that produces immiseration among the rest of society; our model assumes the separateness of politics and economics, and an interaction between separate economic and political systems whereby revenue generation and state capacity are the key interacting variables. Finally, in line with complexity theory, Turchin puts forward multipath modeling with various outcomes (Turchin et al. 2018); in contrast we extend our rather mechanical model linearly into the future.

Our approach follows Turner who develops ‘propositions as “verbal equations” in the sense that one force is seen as a “function” of others’ (Turner, 1995:9). We also follow Collins who has argued that statistics – including descriptive ones - must be translated into words (Collins, 1988:494–511). We do not use multiple regression models, and Turchin also avoids these, but nor do we use statistical path models with variables, while Turchin uses variables for complexity dynamics. Turchin says that elite overproduction, meaning the overproduction of university degrees (Turchin, 2023: xi), with too few economically rewarding positions to match has led to a crisis in the US and elsewhere, but we do not include it. Credential inflation, combined with automation, could have other effects, such as job losses for the middle classes which may, in turn, lead to a dramatic political shift in the future (Collins, 2013). Rather than causing counter-elites to emerge when they cannot obtain the elite jobs they expect, we suggest that credential inflation could lead to political shifts or to conditions of high status and low economic reward among elites.

Bourdieu describes groups with high cultural capital and low economic capital in *Distinction* (Bourdieu, 1984), so this form of stratification is not new. The separation of status and economic reward inherent in the separation of cultural capital and economic capital is a key element in Bourdieu’s theory. High-status but ill-paid social groups have existed for a long time and Bourdieu points to several examples. The presence of these high-status, low-income groups has not been a factor in political instability in the past. Turchin’s ideas about this factor only apply to the past and his forecast is of immanent crisis but we know of no long-term projections of educational credential inflation into the future (though Fig. 3 shows a sharp increase in educational qualifications for China).

Turchin’s modeling consists of what he calls ‘compartments’ and ‘processes’ (2016: 24). Our model is based on the relations between states and markets (Schroeder, 2013). But the language used is also different among various approaches in the social sciences: Some would say ‘factors’ (as in the ‘economic’ factor) and others talk about the ‘primacy’ in the relation between different factors, including in the ‘sources of social power’ (Mann, 1986). Integrating the various ways in which comparative-historical analysis can be systematized to facilitate cumulation and forecasting is still at an early stage (Kreuzer, 2023). Some might regard the project of modeling macro-social patterns as hubris: don’t many factors influence societal development? However, It is unclear why these patterns should be less modellable. Some patterns (such as the size of aging population cohorts) are more predictable than others (GDP) even if uncertainty grows as the model is projected farther into the future. Further, if we think about mitigating climate change, there is a clear need for models..

Readers may regard the model put forward here as too simple to be considered AI. Our response to this is threefold: first, if AI is defined as machines that learn to learn, then our model qualifies, albeit from the low threshold of theories in the domain of macro-social theory. Second, despite our model’s rather low technical sophistication, we build on a strong cumulative tradition in social science, centering on the study of long-term economic and political change. Third, datasets for examining political and economic change must fit the social reality they seek to uncover rather than reflect a perceived need for technical or methodological complexity or the elaborateness of data sources. In our case, the datasets are widely available, and the bottleneck is the absence of high-powered models that are clearly described, especially the relations between different ‘variables’ or parts of social ‘systems’ rather than more complex data and methods (Schroeder, 2023b).

Turchin’s model is aimed at crises, ‘elite overproduction’ (2016: 11, 16–17). He builds on a well-established state breakdown theory in social science that conflict among elites coupled with economic constraints leads to political instability (Collins, 1999: 19–36). Against this, our model posits future economic constraints on state capacity which, if unchecked, will lead – at least in the case of China – to a sharply diminished state capacity to provide essential social services. But the same constraint could also affect India and the US, though not Sweden. A structural-demographic theory or model can, in our view, be taken as a starting point: it sets the boundaries – apart from the growing threat of a potential climate change crisis, which can be added to our model (for example, Schroeder, 2022) – to the conditions for societal development. While Turchin’s model focuses on education and the economic position of elites, our model focuses on the central driving force of the increase in government spending since World War 2: the rising requirements for social welfare spending. This historical change is well-documented (see Tanzi & Schuhknecht, 2000: 6–7, table 1.1 and 1.2, Yörük et al., 2022; see also https://emw.ku.edu.tr/project/project-summary/), though it has not to our knowledge been used for making projections into the future. Of course, not all social spending is state spending; there is always private sector provision of health, education and pensions. But in this case, structural constraints come up against growing economic inequalities in China, India and the US, though again, not in Sweden (see World Inequality Database https://wid.world/, Piketty, 2020; a contrary view for the US is Auten & Splinter, 2024).

Of course, crises and instabilities may not be mutually exclusive, and Turchin’s forecast of a near-term crisis may be more useful for policymaking than our model which is aimed at systematizing social science models that can be built upon. Both will be useful in developing more computationally-driven modeling of societal development that is bound to become more high-powered with AI. One way forward in this regard could be forecasting tournaments, whereby social scientists are provided with historical trend data and their predictions are pre-registered for certain domains, though so far this has only been done for more short-term forecasts (see Grossmann et al., 2023). We have made a start in developing comparable models that can be tested against each other using AI tools. As these tools become more accessible, learning to learn by iteratively testing and comparing models against each other and against the available data sources can contribute to the cumulation of social scientific knowledge.

## Figures and Tables

**Table 1:** Summary of forecasts.

Country	Key Driver	Our prediction (period: 2029–2050)	ClaudeAI Prediction (period: 2022–2050)
China	Rapid decline in working-age population	Fiscal Crisis	Spending needs up, revenue down
India	Slow decline in working age population	Need for higher tax revenue likely	Growth and greater capacity to tax
Sweden	Deficit to cope with high spending has been ongoing	Stable	Stable at high spending/high revenue level
US	Higher spending needs for social security	Stable, but may need political will to cope with spending increase	Steady, some increased costs due to aging

## Data Availability

The data are publicly available, as described in the main text.

## Footnotes

**Endnotes**
1.The WPP displays 10 population projection scenarios and the World Bank documentation does not say which one of the 10 projections it uses. We might guess that the World Bank used the WPP “medium” scenario. In its definition of population scenarios the United Nations says that “The medium scenario projection corresponds to the median of several thousand distinct trajectories of each demographic component derived using the probabilistic model of the variability in changes over time.” (https://population.un.org/wpp/DefinitionOfProjectionScenarios/). This translates into a projection using “medium” levels of fertility, mortality and net migration. The most recent China data are based on the 2020 census and official 2021 estimates (https://population.un.org/wpp/datasources/156). Most recent India data are based on the 2011 census plus official estimates through 2021.(https://population.un.org/wpp/DataSources/356). The most recent Swedish data are based on the 2011 census, also plus official estimates through 2021 (https://population.un.org/wpp/DataSources/752). The most recent US data are from the 2020 census with an official estimate for 2021 (https://population.un.org/wpp/DataSources/840).
